# T-cell dynamics after intracerebral hemorrhage and stroke-associated pneumonia: the vicious cycle of the lung–brain axis

**DOI:** 10.3389/fnins.2026.1953214

**Published:** 2026-10-09

**Authors:** Jingyan Li, Wei Ni

**Affiliations:** Department of Neurosurgery, Huashan Hospital, Fudan University, Shanghai, China

**Keywords:** immunosuppression, intracerebral hemorrhage, lung–brain axis, neuroinflammation, stroke-associated pneumonia, T cells

## Abstract

Spontaneous intracerebral hemorrhage (ICH) is a devastating cerebrovascular disorder associated with high mortality, severe disability, and substantial clinical burden. Stroke-associated pneumonia (SAP), one of the most common infectious complications after stroke, is increasingly recognized as a major contributor to mortality and poor neurological outcomes after ICH. Emerging evidence from studies of stroke-induced immunosuppression and the lung–brain axis suggests that SAP is not merely a secondary pulmonary complication, but rather a multifactorial consequence of central nervous system injury, peripheral immune remodeling, and impaired pulmonary host defense. Among these processes, T cells serve as a critical immunological relay between the central nervous system and peripheral immunity. During the acute phase of ICH, pro-inflammatory T-cell subsets infiltrate the injured brain and amplify neuroinflammation, blood–brain barrier disruption, cerebral edema, and neuronal injury. As the disease progresses, T-cell hyporesponsiveness and suppression of Th1/Th17-mediated antimicrobial immunity may increase increase susceptibility to SAP. Once SAP develops, pulmonary inflammation, microbiota dysbiosis, bacterial products, and systemic inflammatory mediators may further aggravate central immune dysregulation through the lung–brain axis, thereby establishing a vicious cycle involving ICH, T-cell dysfunction, SAP, and secondary brain injury. This review summarizes the temporal dynamics, trafficking patterns, and functional transitions of T-cell subsets after ICH, with particular emphasis on their potential roles in the bidirectional interactions between SAP and the injured brain along through the lung–brain axis. A better understanding of this T-cell-centered network may reveal potential targets for preventing SAP, restoring post-ICH immune homeostasis, and attenuating secondary neurological injury.

## 1 Introduction

Spontaneous intracerebral hemorrhage (ICH) is a common acute cerebrovascular disorder (Wang et al., 2023). According to the 2024 China Stroke Prevention and Treatment Report, intracerebral hemorrhage accounts for approximately 28.7% of incident stroke cases and remains associated with relatively high mortality and disability, imposing a substantial socioeconomic burden. Traditionally, the inflammatory immune response triggered by the extravasation of blood components, cellular debris accumulation, and local tissue necrosis after hematoma formation has been regarded as a key pathological determinant of the severity of secondary brain injury and long-term outcome (Ohashi et al., 2023). Secondary injury after ICH is driven by multiple interconnected processes, including thrombin toxicity, hemoglobin/heme/iron-mediated injury, oxidative stress, blood-brain barrier (BBB) disruption, cerebral edema, and innate neuroinflammation (Keep et al., 2014). Within this framework, microglia/macrophages and neutrophils have long been considered the principal inflammatory effectors, whereas T cells received comparatively less attention in earlier studies (Taskin and Yao, 2025). However, accumulating evidence now suggests that acute-phase T cells can contribute to pro-inflammatory and tissue-injurious responses, whereas regulatory T cells (Tregs) may suppress microglia-mediated inflammation and exert neuroprotective effects (Zhao et al., 2014).

In parallel, more recent studies indicate that central and peripheral immunity after ICH undergoes a dynamic and compartment-specific remodeling, in which central inflammatory activation may overlap temporally with peripheral immunosuppression (Yu et al., 2021). During this process, T cells serve as an important interface between innate and adaptive immunity and participate throughout the shift from early inflammatory activation to subsequent immune suppression. T cells therefore contribute not only to amplification of central neuroinflammation during the acute stage of ICH, but also to the development of systemic immune dysfunction and post-stroke infectious susceptibility.

During the acute phase, γδ T cells, Th1-like effector T cells, and a subset of Th17 cells enter the injured brain and release IL-17, IFN-γ, TNF-α, and related inflammatory mediators, thereby aggravating cerebral edema, BBB disruption, and neuronal injury. As the disease progresses, the immune response shifts from a pro-inflammatory state toward immunosuppression. At this stage, regulatory T cells (Tregs) become the predominant T-cell subset within the central nervous system, where they facilitate hematoma clearance, restrain inflammatory injury, and promote neural repair. In parallel, peripheral immunosuppression is characterized by lymphopenia, impaired T-cell function, weakened antimicrobial immunity, and increased susceptibility to pulmonary infection. The temporal dynamics and functional roles of distinct T-cell subsets after ICH are summarized in Table 1.

**TABLE 1:** Temporal dynamics and functional roles of brain-infiltrating T-cell subsets after intracerebral hemorrhage.

Phases of ICH	Types of T cells	Effects on ICH	Mechanisms
Acute phase (0–3 days after ICH)	γδ T cells/γδT17	Detrimental	Activated downstream of microglia/macrophages by IL-1β and IL-23; produce IL-17A and amplify BBB dysfunction, perihematomal edema, and neuronal injury.
	Th1-related CD4+ T cells	Potentially detrimental	IFN-γ- and TNF-α-centered signaling enhances antigen presentation, myeloid activation, endothelial inflammation, and leukocyte engagement with the BBB.
	Th17-related CD4+ T cells	Detrimental	IL-17 signaling promotes chemokine production, neutrophil recruitment, endothelial dysfunction, BBB disruption, and edema progression.
	CD4+ T cells	Detrimental	Perihematomal infiltration is associated with edema progression and neurological deterioration; IL-17A- and TRAIL-DR5-related endothelial injury may worsen barrier leakage.
	CD8+ T cells	Potentially detrimental	May contribute to BBB and parenchymal injury through perforin- and granzyme B-related cytotoxicity, although direct ICH evidence remains limited.
	Pan-T-cell activation	Detrimental	TCR-related signaling amplifies pro-inflammatory cytokine production, leukocyte infiltration, cerebral edema, and neurological deficits.
Subacute/recovery phase (3–14 days after ICH)	Tregs	Protective	Suppress ongoing secondary inflammation, restrain effector T cells, infiltrating myeloid cells, and microglia, and reduce harmful TNF-α/IFN-γ-related signaling.
	Tregs	Protective	Support tissue repair and neurovascular unit homeostasis; IL-10-, TGF-β-, and FOXP3-related regulation may reinforce post-ICH immune braking.
	Treg-microglia crosstalk	Protective	Promotes the shift of microglia toward a repair phenotype; Treg-derived osteopontin enhances microglial repair activity and favors white matter/myelin regeneration.
	Tregs and BBB repair	Protective	CCR5-mediated docking to injured vessels and suppression of neutrophil-associated MMP-9 responses help stabilize the BBB and limit inflammatory extravasation.
	Treg/Th17 balance	Protective when shifted toward Treg	Regulated in part by dendritic cell-TLR signaling; TLR4/TLR2-driven DC maturation lowers the Treg/Th17 ratio, whereas TLR9-IDO1 signaling shows the opposite tendency.

The lung–brain axis refers to a bidirectional communication network through which the respiratory system and the central nervous system (CNS) interact via neural, immune, endocrine, metabolic, microbial, and gas-exchange–dependent pathways. Rather than functioning as two anatomically isolated organs, the lung and brain dynamically influence each other under both physiological and pathological conditions. Acute brain injury can disrupt autonomic and neuroendocrine homeostasis, induce systemic immune alterations, and promote pulmonary endothelial and epithelial injury, thereby increasing susceptibility to neurogenic pulmonary edema, acute lung injury, and pneumonia. Conversely, pulmonary inflammation may transmit inflammatory or neural signals to the CNS, leading to blood–brain barrier dysfunction, microglial activation, white matter injury, and neuronal damage (Ziaka and Exadaktylos, 2024). Evidence from experimental models outside the specific ICH setting further supports this bidirectional paradigm. Traumatic brain injury has been shown to promote the release of extracellular vesicles containing inflammasome-related proteins into the circulation, subsequently activating pulmonary inflammasomes and inducing acute lung injury, providing a mechanistic example of brain-to-lung signaling (Kerr et al., 2018). In the opposite direction, pulmonary inflammatory stimuli and ventilator-induced lung injury have been reported to promote CNS inflammation and neuronal injury, partly through circulating IL-6–dependent signaling (Witzenrath and Kuebler, 2021). More recently, experimental acute lung injury was found to induce cerebral white-matter demyelination through a cervical vagus nerve-dependent pathway, further supporting the possibility that lung-derived pathological signals can reach the brain through defined neuroanatomical circuits (Xu et al., 2025). Together, these findings indicate that lung–brain communication is reciprocal, multimodal, and may contribute to generating self-amplifying inflammatory responses.

Compared with ischemic stroke and traumatic brain injury, the lung–brain axis remains insufficiently characterized in intracerebral hemorrhage (ICH). Nevertheless, clinical studies identify stroke-associated pneumonia (SAP) as a frequent complication of ICH and have associated pulmonary infection with increased mortality, neurological disability, readmission, and unfavorable functional outcomes (Ji et al., 2014). Therefore, elucidating how ICH-induced neurological and immune disturbances predispose the lung to infection, and how subsequent pulmonary inflammation feeds back to aggravate secondary brain injury, is essential for understanding ICH progression and identifying potential targets for preventing systemic complications and improving neurological recovery.

Collectively, post-stroke immunosuppressive syndrome and the lung-brain axis are increasingly recognized as central concepts for understanding why SAP is not merely a secondary complication of ICH, but may also represent a consequence of systemic immune reprogramming and a potential amplifier of ongoing brain injury. Therefore, a systematic evaluation of the temporal dynamics of T-cell responses after ICH, their injurious and protective roles, and their roles in the bidirectional interactions between ICH and SAP is important for clarifying mechanisms of secondary brain injury, refining infection prevention strategies during the peri-stroke period, and identifying rational targets for precision immunomodulation. This review therefore focuses on the transition of T-cell responses from inflammatory activation to immunosuppression after ICH, the neuroendocrine basis of stroke-induced immunesuppression, and the proposed bidirectional feedback loop through which T-cell dysfunction may contribute to SAP and, in turn, pulmonary inflammation may aggravate secondary brain injury via the lung–brain axis.

## 2 Acute initiation of central inflammation and T-cell recruitment after ICH

### 2.1 DAMP-driven innate immune activation and adaptive T-cell engagement after ICH

Within minutes to hours after ICH, the perihematomal brain develops sterile inflammation (Ziai, 2013). Hematoma expansion, mechanical compression, and local ischemia and hypoxia injure neurons and glial cells, leading to the release of DAMPs such as HMGB1, ATP, DNA fragments, myelin fragments, hemoglobin, heme, and iron (Chen R. et al., 2022). These damage signals activate microglia, peripheral macrophages, and cerebrovascular endothelial cells through Toll-like receptors (TLR), NOD-like receptors, and other pattern recognition receptors. This signaling activates downstream inflammatory pathways, including NF-κB, and promotes the production of pro-inflammatory mediators, including TNF-α, IL-1β, IL-6, and IL-23, leading to a series of inflammatory reactions such as neuronal damage and cerebral edema (Fang et al., 2013). Neutrophils and microglia are the earliest cell groups to participate in the response, and the changes in the inflammatory microenvironment caused then create the necessary conditions for T cells to enter the CNS (Lei et al., 2022). Importantly, T-cell involvement after ICH should not be regarded solely as a secondary consequence of innate immune activation and BBB disruption. Evidence from experimental ICH indicates that antigen-dependent T-cell receptor (TCR) signaling can also contribute to T-cell activation, and inhibition of this pathway attenuates T-cell-mediated neurotoxicity and pulmonary inflammatory responses (Xiu et al., 2025; Chen et al., 2025). Supporting evidence from related experimental stroke models further suggests that CNS antigen-specific T-cell responses may influence neurological injury (Jin et al., 2018). Thus, innate inflammatory signaling, BBB disruption, and adaptive TCR-dependent activation may collectively shape T-cell recruitment and effector responses after ICH.

### 2.2 Pro-inflammatory T-cell responses in acute ICH promote BBB disruption, cerebral edema, and neuronal injury

The main role of T cells in the acute phase of ICH is generally pro-inflammatory. After ICH, innate immune cells in the perihematomal region are rapidly activated, and TCR activation of brain-infiltrating T cells and can promote the release of pro-inflammatory cytokines and further amplify this response. CNS inflammation models suggest that microglia can activate γδ T cells via IL-1β and IL-23 and promote their IL-17-related effects; in the ICH model, T-cell activation is closely related to infiltration of other leukocytes, cerebral edema, and neurological deterioration. Therefore, acute-phase T cells are not simply bystander infiltrates, but more like an important adaptive immune node connecting innate inflammation amplification and secondary brain injury (Xiu et al., 2025).

IL-23 can drive γδ T cells to produce IL-17, and IL-1β and IL-23 can further promote their differentiation or polarization toward a γδT17 phenotype. Subsequently, IL-17 can act on endothelial cells, astrocytes, and microglia/macrophages in the brain, inducing the expression of chemokines, pro-inflammatory mediators, and matrix metalloproteinases, promoting the recruitment of neutrophils, monocytes, and more lymphocytes into the perihematomal area. Inhibiting TCR activation after ICH onset can reduce the activation of brain-infiltrating T cells and the levels of pro-inflammatory cytokines in the brain, and reduce the infiltration of other white blood cells, cerebral edema, and long-term neurological damage; related experimental evidence further suggests that suppression of T-cell-associated inflammatory responses may attenuate white matter injury (Chen et al., 2025). In addition to CD4+ T-cell-mediated cytokine damage, CD8+ T cells can also directly mediate target cell damage through perforin/granzyme B-related mechanisms (Shi et al., 2023).

Blood-brain barrier disruption is a key component of secondary brain injury caused by ICH and one of the important targets for the damaging effects of T cells. Studies of the cerebrovascular endothelium and experimental stroke indicate that IL-17A can directly impair the cerebral microvascular endothelial barrier. IL-17A induces reactive oxygen species production in brain endothelial cells, activates the endothelial actomyosin contractile machinery, and increases interendothelial gaps and barrier permeability. It can also disrupt the distribution or reduce the expression of tight-junction-associated proteins, including ZO-1, occludin, and claudin-5 (Rahman et al., 2018). In parallel, IL-17A acts synergistically with TNF-α to enhance the expression of neutrophil-attracting CXC chemokines, particularly CXCL1, in astrocytes and other neurovascular cells, thereby promoting neutrophil recruitment and amplifying post-stroke inflammation (Mathias et al., 2012). In ICH and related hemorrhagic stroke models, brain-infiltrating T-cell responses are associated with endothelial injury, increased BBB permeability, plasma protein extravasation, perihematomal edema, and neurological deterioration. Pharmacological restriction of lymphocyte trafficking or inhibition of T-cell receptor activation has been reported to reduce vascular permeability, inflammatory-cell infiltration, and cerebral edema after experimental ICH (Rolland et al., 2013). Furthermore, astrocyte-derived CCL5 promotes the recruitment and activation of CD8+ T cells after hemorrhagic stroke, while activated CD8+ T cells express perforin and granzyme B and contribute to endothelial cell death and BBB disruption, indicating a CCL5-mediated astrocyte–CD8+ T-cell cytotoxic axis within the injured neurovascular unit (Zhou et al., 2024).

In terms of neuronal and white matter damage, different subpopulations of T cells in the acute phase have both indirect and direct damaging effects. Indirectly, T-cell-related IFN-γ, TNF-α, IL-17, and IL-1β signaling can amplify microglial/macrophage inflammatory responses and enhance the production of ROS, MMPs, and other injury-associated mediators, all of those aggravating neurovascular unit imbalance, cerebral edema, and secondary parenchymal damage. Directly, CD4+ T-cell-related pro-apoptotic signals participate in endothelial cell death, and the perforin/granzyme B axis of CD8+ T cells can also mediate target cell damage; at the same time, regulating CD4+ T-cell differentiation can reduce white matter damage after ICH.

## 3 Alterations in T-cell composition during the subacute phase are associated to changes in immune responses

### 3.1 Compartment-specific T-cell remodeling during the subacute phase of ICH

Approximately 3–14 days after ICH, the T-cell response does not undergo a simple, uniform transition from a pro-inflammatory state to immunosuppression. Instead, it exhibits marked compartment-specific remodeling between the central nervous system (CNS) and the periphery (Zhang et al., 2017). Within the CNS, T-cell infiltration remains active during the first week after ICH. Experimental studies have shown that brain-infiltrating T cells begin to increase as early as day 1 and reach a peak at approximately days 4–5. Shi et al. (2023) reported that T-cell infiltration in the hemorrhagic hemisphere peaked on day 5, in parallel with increased blood–brain barrier (BBB) permeability. CD4+ T cells represent one of the predominant T-cell populations in the brain during this period. Enrichment of CD4+ T cells has been observed both in perihematomal tissue obtained within 72 h from patients with ICH and in experimental mouse models, with CD4+ T cells outnumbering CD8+ T cells. These cells also exhibit increased expression of CD69 and IL-17 (Xie et al., 2023; Xiu et al., 2025). Single-cell transcriptomic analysis performed on day 3 after experimental ICH further demonstrated that brain-infiltrating CD4+ T cells displayed transcriptional features associated with inflammation, apoptosis, leukocyte migration, and IL-17 signaling. Functionally, these cells may contribute to BBB disruption, perihematomal edema, and cerebrovascular endothelial injury through IL-17- and TRAIL–DR5-related pathways.

Other T-cell subsets also undergo distinct compositional changes during the subacute phase. γδ T cells constitute an important component of early ICH-associated neuroinflammation. Although brain-infiltrating γδ T cells remain detectable during the subacute phase, their relative proportion within the brain gradually declines (Zhong et al., 2016). At the same time, regulatory T cells (Tregs) begin to emerge within the CNS. Clinical studies have reported an increased proportion of circulating Tregs on days 3 and 7 after ICH, accompanied by elevated levels of IL-10 and TGF-β (Shi et al., 2015). Other studies have also suggested that Tregs can be recruited to perihematomal tissue during the first week after ICH (Deng et al., 2023). Functionally, Tregs may suppress the release of pro-inflammatory cytokines, attenuate BBB injury, and promote a more reparative immune environment. This shift in T-cell composition from a γδ T-cell-dominated inflammatory response toward increasing Treg involvement may therefore contribute to the transition of the CNS immune response from an inflammation-promoting phase toward a phase characterized increasingly by immune regulation and neural repair.

In contrast to the persistent recruitment of T cells within the CNS, the peripheral T-cell compartment undergoes substantial compositional remodeling during the same period. Zhang et al. longitudinally assessed patients with ICH at admission and on days 3 and 14 and found that total lymphocyte counts and CD4+ T-cell numbers decreased after ICH, reached their lowest levels at approximately day 3, and remained reduced at day 14 (Zhang et al., 2018). CD8+ T cells also showed a declining trend. More importantly, patients who developed infections during hospitalization had significantly lower numbers of CD4+ T cells, CD8+ T cells, and NK cells throughout days 1–14 than those who remained infection-free. Similar reductions in peripheral T and NK cells have also been observed in experimental ICH models (Cheng et al., 2022). Thus, while T-cell infiltration persists within the CNS, the conventional peripheral effector T-cell pool undergoes sustained depletion, illustrating a clear divergence between central and peripheral immune compartments.

The composition of the remaining peripheral CD4+ T-cell population is also altered. Shi et al. (2015) reported that the proportion of circulating CD4+CD25^highCD127^low/−FoxP3+ Tregs was increased on days 3 and 7 after ICH, with higher Treg proportions observed in patients with more severe neurological impairment. These changes were accompanied by increased circulating IL-10 and TGF-β. Direct evidence from ICH studies remains insufficient to establish a definitive Th1-to-Th2 transition. However, longitudinal studies in ischemic stroke have shown that Th1 and Th17 cells increase around day 3 and subsequently decline by day 7, whereas Th2 cells display an opposite temporal pattern (Yu et al., 2022).

These compositional alterations are accompanied by impaired T-cell function. Experimental ICH studies have demonstrated increased PD-1 expression on peripheral T-cells and NK-cells, together with a reduction in their cell numbers. Blockade of PD-1 signaling attenuated the contraction of the T- and NK-cell pools and was accompanied by a reduction in pulmonary bacterial burden (Illanes et al., 2011; Zhang et al., 2024). Taken together, the subacute phase of ICH is characterized by a distinct central–peripheral immune divergence: the CNS continues to harbor CD4+, γδ T-cell, and Th17-associated inflammatory populations while progressively acquiring Treg-mediated regulatory responses, whereas the peripheral compartment exhibits sustained depletion of CD4+ and CD8+ effector T cells, relative enrichment of Tregs, and reduced T-cell effector function.

Ultimately, this remodeling of peripheral T-cell composition and function results in a reduced pool of effector lymphocytes available for pathogen clearance, together with impaired antimicrobial cellular immunity. Consistent with this pattern, patients with ICH who develop infectious complications show more pronounced deficiencies in CD4+ T cells, CD8+ T cells, and NK cells. Therefore, the subacute peripheral immune phenotype, characterized by effector T-cell depletion, relative enrichment of regulatory components, and suppression of T-cell function, may establish an important immunological background that predisposes patients with ICH to the development of stroke-associated pneumonia (SAP).

### 3.2 Functions of T-cell subsets during the subacute phase of ICH

#### 3.2.1 Tregs serve as a key T-cell subset mediating neuroprotective and anti-inflammatory effects during the subacute phase of ICH

Unlike pro-inflammatory T cells in the acute phase, regulatory T cells (Tregs) often play immunosuppressive and tissue-protective roles during the recovery phase of brain injury. Although evidence on the precise time course of Tregs in the brain after ICH is limited, ischemic stroke studies have shown that Tregs in the brain can continue to accumulate during the chronic recovery phase and promote neurological recovery (Shang et al., 2024). Alterations in the Treg/Th17 balance after ICH may arise in part from remodeling of the DC-T-cell axis. Li et al. (2024) found that TLR4/TLR2-mediated DC maturation after ICH reduces central and peripheral Treg/Th17 ratios and aggravates neuroinflammation, while TLR9 exerts opposite regulatory effects on Treg/Th17 balance through an IDO1-related mechanism. This study suggests that the relative increase in Tregs after ICH is not simply caused by a reduction in effector T cells, but may be regulated by the DC-TLR9-IDO1 metabolic immune axis. However, general immunological studies have shown that glucocorticoids can upregulate FOXP3 expression and enhance Treg-related functions; therefore, HPA axis activation after ICH may inhibit effector T cells such as Th1/Th17 while also promote Treg stability or enhance their suppressive function through GR-FOXP3-related mechanisms. In addition, IL-10 and TGF-β-related anti-inflammatory milieu may also be involved in the enhanced Treg response after ICH. Ischemic stroke models show that IL-33 can expand FoxP3+Tregs in the brain through ST2 receptors, accompanied by increased levels of IL-10 and TGF-β1; in the chronic stroke stage, Treg expansion in the brain also relies on IL-2, IL-33, 5-HT and TCR recognition (Liu et al., 2020).

The protective effects of Tregs are mainly reflected in limiting secondary inflammation and promoting repair. Studies on ischemic stroke have shown that Tregs can limit the excessive activation of effector T cells, infiltrating myeloid cells and microglia, and reduce harmful inflammatory signals such as TNF-α and IFN-γ; among these mechanisms IL-10 signaling is particularly critical for its immunoregulatory effect. At the vascular interface, Tregs may also attenuate early post-stroke BBB disruption by interacting with the injured vessel wall, thereby limiting further extravasation of inflammatory cells (Li et al., 2017). During the recovery period, Tregs in the brain can also inhibit neurotoxic astroglial reactions through repair-related molecules such as amphiregulin and promote neurological recovery. Therefore, Tregs may also exert neuroprotective effects during the subacute phase of ICH by suppressing ongoing myeloid inflammation, improving neurovascular unit homeostasis, and promoting tissue repair.

In summary, Tregs may exert compartment-dependent effects during the subacute phase after ICH. Within the CNS, their immunosuppressive activity likely contributes to limiting persistent neuroinflammation, stabilizing the neurovascular unit, and promoting tissue repair. In the periphery, however, a prolonged or excessively suppressive Treg-associated phenotype may participate in post-stroke immunodepression and impaired antimicrobial defense. Evidence supporting this latter effect is derived mainly from ischemic stroke and infection models rather than ICH-specific studies, and Treg expansion alone has not been established as a direct cause of pneumonia (Lukasik et al., 2023). Instead, Treg-mediated suppression likely acts together with lymphopenia, impaired Th1/IFN-γ responses, and neuroendocrine stress signaling to increase susceptibility to post-stroke infectious complications.

#### 3.2.2 Functions of other T-cell subsets during subacute phase of ICH

During the recovery period after ICH, the transition of microglia from a pro-inflammatory to a repair phenotype is considered an important marker of inflammation resolution and tissue remodeling, where Tregs play an important role. However, the other T cells subsets also participate in the subacute phase immune response during the subacute phase.

During the early subacute phase of ICH, effector T-cell subsets exhibit marked functional divergence between the CNS and peripheral compartments. In experimental ICH, γδ T cells remain prominent within the hemorrhagic brain, reaching high levels around day 4. Macrophage-derived IL-23 stimulates γδ T-cell production of IL-17, thereby amplifying IL-1β- and TNF-α-associated inflammation, cerebral edema, and neurological injury (Zhong et al., 2016). Peripheral γδ T-cell numbers can also increase by day 3 in experimental ICH (Gao et al., 2014), although their specific contribution to systemic antimicrobial immunity remains insufficiently defined. CD4+ effector T cells accumulate in perihematomal tissue from approximately 24 h and become most prominent around days 4–5. These cells display activated CD69+/IL-17+ phenotypes and contribute to BBB disruption, endothelial injury, and perihematomal edema through IL-17- and TRAIL–DR5-related mechanisms (Xie et al., 2023). In contrast, circulating CD4+ T cells contract by day 3 and remain particularly deficient in patients who develop infection; increased PD-1 expression on day 3 further indicates functional suppression. CD8+ T-cell infiltration is less prominent than CD4+ T-cell infiltration, but by day 3 reactive astrocyte-derived CCL5 can recruit CD8+ T cells into the hemorrhagic brain, where perforin/granzyme B-dependent endothelial injury contributes to BBB disruption (Cheng et al., 2022; Zhou et al., 2024). Peripheral CD8+ T cells concurrently decline and show increased PD-1 expression. Th17 cells remain elevated in the peripheral circulation during days 1–4 and approach control levels by day 7, consistent with attenuation of the Th17 inflammatory program.

## 4 The main mechanism of T cells component remolding in ICH subacute phase

Although the acute phase of ICH is dominated by focal pro-inflammatory reactions, as the disease progresses, the body does not continue to maintain a highly inflammatory state, but gradually enters an immunosuppressive phase characterized by reduced peripheral immune responsiveness and increased susceptibility to infection. Clinical studies have found that patients with ICH often experience a decrease in peripheral lymphocyte counts after onset, accompanied by splenic contraction, decreased monocyte antigen presentation capacity, and weakened T-cell and natural killer (NK) cell functions. Current evidence suggests that the shift of peripheral immunity from pro-inflammatory to immunosuppressive after ICH is mainly mediated through three pathways: (1) Hypothalamic-pituitary-adrenal axis (HPA) (2) Sympathetic-adrenal axis (3) Vagus nerve-cholinergic pathway. The major neuroendocrine mechanisms underlying post-ICH immunosuppression are illustrated in Figure 1.

**FIGURE 1:**
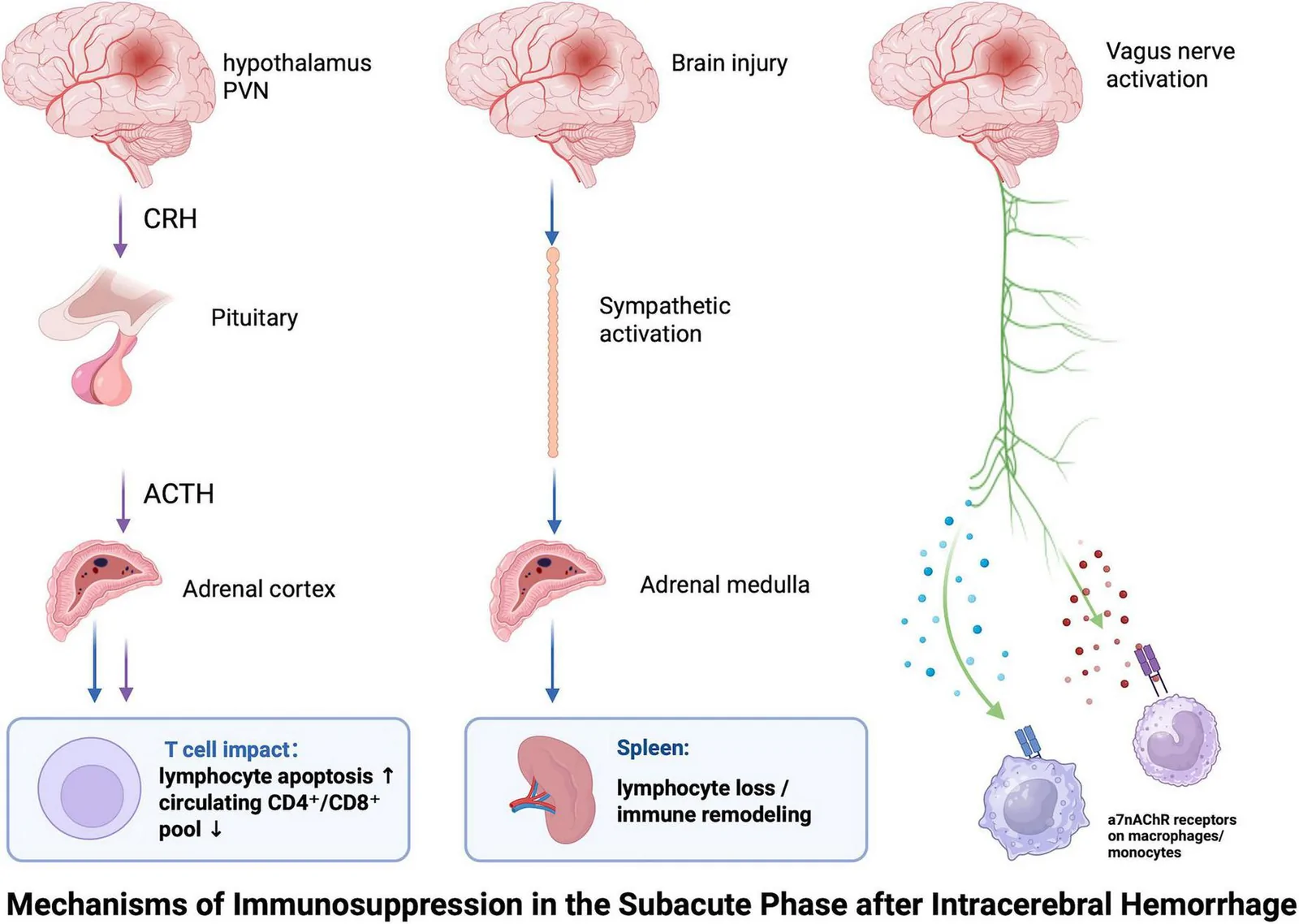
Mechanisms underlying immunosuppression during the subacute phase after intracerebral hemorrhage: brain injury following intracerebral hemorrhage activates multiple neuroendocrine and autonomic pathways that reshape systemic immune responses. (1) Activation of the hypothalamic–pituitary–adrenal axis promotes the sequential release of corticotropin-releasing hormone (CRH) and adrenocorticotropic hormone (ACTH), followed by glucocorticoid secretion from the adrenal cortex. (2) Glucocorticoid signaling alters T-cell polarization and effector activity, suppresses cytokine signals required for the maintenance of pro-inflammatory T-cell responses, and relatively favors FOXP3^+^ regulatory T-cell-associated immunoregulatory pathways. (3) Sympathetic activation stimulates the adrenal medulla and peripheral sympathetic terminals, promoting catecholamine release, splenic lymphocyte loss, and systemic immune remodeling. Vagal activation further engages the cholinergic anti-inflammatory pathway, in which acetylcholine acts on α7 nicotinic acetylcholine receptors expressed by monocytes and macrophages, thereby attenuating innate inflammatory responses. Together, activation of the hypothalamic–pituitary–adrenal axis, sympathetic nervous system, and vagal anti-inflammatory pathway contributes to peripheral immunosuppression and increased susceptibility to infection after intracerebral hemorrhage. Created by the authors using BioRender.com.

### 4.1 HPA axis: glucocorticoid-mediated lymphopenia and suppression of T-cell responses

After ICH, hematoma compression, increased intracranial pressure, and brain injury-related stress can activate the hypothalamic-pituitary-adrenal axis and promote the release of ACTH and glucocorticoids; clinical studies have also shown that patients with ICH may exhibit elevated serum cortisol levels early after admission, and this change is associated with adverse outcomes (Chen X. G. et al., 2022). In the periphery, elevated glucocorticoids reshape immune cell transcriptional programs mainly through the glucocorticoid receptor (GR), broadly inhibit pro-inflammatory transcriptional signaling, and weaken T-cell activation, proliferation, and pro-inflammatory responses such as IFN-γ production, inhibiting Th1/Th17-like responses (Mracsko et al., 2014). In patients with acute cerebrovascular disease, this neuroendocrine stress response is often accompanied by early lymphopenia and cellular immune hyporesponsiveness. In addition, research by Karagiannidis et al. (2004) showed that glucocorticoids can upregulate FOXP3 expression and enhance Treg-related functions; subsequent studies further suggested that part of their anti-inflammatory effect depends on FoxP3+ Tregs and may be related to miR-342-related mechanisms. Therefore, peripheral immunity after ICH gradually shifts from a Th1/Th17/γδT-like pro-inflammatory state in the acute phase to a state characterized by T-cell hyporesponsiveness and relative Treg predominance and Treg is relatively dominant, and the HPA axis-glucocorticoid pathway may be one of the important driving factors.

### 4.2 Sympathetic-adrenal medullary axis: catecholamine-driven peripheral immune remodeling

In a study of the stroke-associated sympatho-adrenal axis, Prass et al. (2003) found in MCAO mice that animals developed spontaneous pulmonary and systemic bacterial infections within days of experimental stroke, accompanied by widespread lymphocyte apoptosis, and a defective IFN-γ response. Adoptive transfer of T/NK cells or early administration of IFN-γ after stroke reduced the bacterial load, while blocking the sympathetic nervous system restored the IFN-γ response and reduced infection, suggesting that excessive activation of the sympathetic nervous system and its mediated catecholamine response are important mechanisms of impaired antimicrobial immunity after stroke (Anrather, 2016).

Sympathetic nerve excitation after stroke can promote the release of catecholamines from the adrenal medulla and sympathetic nerve terminals, and these signals regulate peripheral immune cell function mainly through β-adrenergic receptors. Existing research suggests that norepinephrine/β2 adrenergic signaling can reduce pro-Th1 signals such as IL-12 and alter T-cell differentiation by inhibiting dendritic cell NF-κB and AP-1 related pathways, weakening the antibacterial cellular immune response dominated by IFN-γ; this pattern is consistent with the phenotype of peripheral immunity shifting from a pro-inflammatory/antibacterial state to an immunosuppressive state after stroke.

The spleen is a key peripheral immune organ in this process which receives rich sympathetic innervation and stores large numbers of T cells, B cells, monocytes, and other myeloid cells, making it highly sensitive to post-stroke neuroendocrine signals. Yan and Zhang (2014) observed in MCAO rats that serum metanephrine (MN) and normetanephrine (NMN) levels increased, spleen volume decreased, IFN-γ decreased and IL-10 increased after stroke; after propranolol treatment, IFN-γ increased, whereas IL-10, MN and NMN decreased, while spleen volume increased, suggesting that β-adrenergic signaling is involved in splenic atrophy and Th1 response suppression after stroke.

In addition to T cells, poststroke sympathetic activation impairs early antimicrobial defense mediated by splenic B cells. McCulloch et al. (2017) found that experimental stroke can lead to rapid loss of B cells in the splenic marginal zone, reduced antigen capture capacity, and reduced circulating IgM levels, accompanied by spontaneous pulmonary bacterial infection; patients with acute ischemic stroke also experience reduced IgM levels, and the reduction is more obvious in infected patients. β-adrenergic blockade protects splenic marginal zone B cells, restores IgM levels, and reduces bacterial burden in the lungs, suggesting that susceptibility to infection after stroke arises not only from a defective T/NK cell IFN-γ response but is also associated with impairment of splenic B cells and early humoral immunity.

The HPA axis and glucocorticoids are also involved in post-stroke immune changes. A review by Schulze et al. (2014) pointed out that elevated catecholamines and glucocorticoids after stroke can induce peripheral immune changes, and this stress-induced immunosuppression has certain commonalities in acute diseases such as stroke, trauma, myocardial infarction, and surgical trauma. However, the experimental results of Prass/Dirnagl et al. suggest that HPA axis blockade mainly improves lymphopenia and monocyte inactivation, while β-receptor blockade is more critical for restoring IFN-γ responses, reducing bacterial infection, and improving mortality. Therefore, in the mechanistic chain of susceptibility to infection after stroke, the SNS-catecholamine-β-adrenergic receptor pathway may be one of the core axes connecting brain injury, splenic immune remodeling, and peripheral antimicrobial immune dysfunction.

It should be noted that although β-receptor blockade has a relatively consistent immunoprotective effect in animal experiments, clinical evidence is still inconsistent. A historical cohort study by Maier et al. (2015) showed that β-blockade therapy did not reduce the risk of post-stroke pneumonia but was associated with a reduced risk of urinary tract infection. Subsequent systematic reviews and meta-analyses also failed to confirm that β-blockade significantly prevents stroke-related infections, pneumonia, or sepsis, and suggested that different stroke types, severity, drug selection, and comorbidities may contribute to significant heterogeneity (Yang et al., 2022). The correlation between sympathetic activation and the occurrence of SAP remains to be further studied.

### 4.3 Vagus nerve-cholinergic anti-inflammatory pathway: suppression of monocyte/macrophage pro-inflammatory output indirectly reshapes T-cell polarization

A review by Kang K. et al. (2024) on autonomic dysfunction after ICH pointed out that sympathetic overactivation and abnormal parasympathetic/vagal regulation may occur after ICH and are associated with clinical problems such as neurological outcomes, cardiac complications, blood pressure fluctuations, immunosuppression, and infection. Therefore, modulating autonomic nervous function, especially inhibiting excessive sympathetic responses or enhancing vagus nerve-related anti-inflammatory regulation, may become an important strategy for modulating systemic inflammation and secondary complications after ICH.

The cholinergic anti-inflammatory pathway mainly regulates the inflammatory output of myeloid cells through the vagus nerve-related neuro-immune reflex. Classic studies have shown that vagus nerve stimulation can attenuate systemic inflammatory responses induced by endotoxin (Borovikova et al., 2000). Subsequent studies demonstrated that α7nAChR is an important receptor for cholinergic anti-inflammatory effects, and de Jonge et al. (2005) further suggested that this pathway can inhibit macrophage activation through JAK2–STAT3 signaling. At the level of T-cell immunity, the cholinergic anti-inflammatory pathway is more likely to indirectly affect T-cell polarization by changing the myeloid cytokine environment. Th1 differentiation is centered on the IL-12-STAT4/T-bet axis; Th17 and γδT17 responses are supported by myeloid-derived factors such as IL-1β, IL-6, and IL-23. γδT17 cells can rapidly produce IL-17 and amplify subsequent inflammatory responses when stimulated by IL-1β and IL-23. Therefore, it can be speculated that if α7nAChR activation reduces the pro-inflammatory output of monocytes/macrophages and microglia/macrophages, it may indirectly weaken the formation and amplification of the Th1/Th17/γδT17 axis.

In an ICH model, Su et al. (2022) further provided direct experimental evidence for α7nAChR-related mechanisms. Their research showed that the α7nAChR agonist PNU-282987 can reduce inflammatory mediators such as MCP-1, IL-1β, MMP-9, TNF-α, HMGB1 and TLR2 after ICH, promote the polarization of macrophages/microglia toward a CD206^+^ anti-inflammatory phenotype, and improve BBB tight junction protein expression, cerebral edema, neurological function and cardiac function (Su et al., 2022). This study suggests that enhanced α7nAChR-mediated cholinergic anti-inflammatory signaling after ICH suppresses myeloid inflammatory responses and may indirectly affect subsequent T-cell-mediated immune polarization by reducing the pro-inflammatory cytokine milieu.

### 4.4 Multiple converging factors drive changes in T-cell subset composition

The first level is direct neuroendocrine inhibition of T-cell activation. The HPA axis releases glucocorticoids and the sympatho-adrenal axis releases catecholamines, both of which inhibit T-cell IL-2 production, clonal expansion, and IFN-γ release, leading to a decrease in Th1 responses. Studies by Prass et al. (2003) and Mracsko et al. (2014) support the role of the HPA axis and SNS in T-cell immunosuppression after stroke.

The second level is the functional changes in antigen-presenting cells. In the early stage of ICH, myeloid cells release IL-23, which drives γδ T cells to produce IL-17A; subsequently, DC function and TLR signaling are altered, affecting Treg/Th17 balance. Zhong et al. (2016) demonstrated that macrophage-derived IL-23 can drive the γδ T cell-IL-17A pro-inflammatory axis; Li et al. (2024) showed that DC and TLR-related signals can regulate the Treg/Th17 ratio.

The third layer is the shift in the cytokine environment from pro-inflammatory to anti-inflammatory. In the acute phase, pro-inflammatory signals such as IL-1β, IL-6, IL-12, IL-23, and TNF-α are dominant, favoring Th1/Th17/γδT17 response; after entering the immunosuppressive phase, IL-10, glucocorticoids, and catecholamine-related signals are relatively enhanced, prompting the Treg/Th2 response. Regarding the biologic characteristics of IL-17, a review by Mills (2023) pointed out that IL-17A/F can be produced by CD4+ T cells, CD8+ T cells, γδ T cells and other immune cell subsets, and is often driven by IL-1β and IL-23 and participates in barrier inflammation and neutrophil recruitment.

## 5 Post-ICH immunosuppression and increased susceptibility to SAP

### 5.1 SAP is one of the most important infectious complications after ICH

Stroke-associated pneumonia (SAP) usually refers to a pulmonary infection that occurs within 7 days after stroke and is one of the most common and clinically significant infectious complications after acute stroke (Meisel et al., 2026). Existing guidelines and systematic reviews show that the overall incidence of SAP is approximately 12%, but may fluctuate significantly across different study cohorts and diagnostic criteria; its occurrence is closely associated with increased in-hospital mortality, prolonged hospitalization, and worsening of functional and long-term outcomes (Meisel et al., 2026). Clinically, increasing age, impaired consciousness, swallowing dysfunction, mechanical ventilation, diabetes, and a greater comorbidity burden are all considered to be associated with an increased risk of SAP (Ahmad et al., 2024). In terms of etiology, the common causative bacteria of stroke complicated by pneumonia are mainly Gram-negative bacteria, including *Klebsiella pneumoniae*, *Pseudomonas aeruginosa*, and *Escherichia coli* (Aboulfotooh et al., 2024).

However, traditional risk factors such as aspiration, bed rest, and mechanical ventilation alone are not sufficient to explain the occurrence of SAP. More and more studies suggest that peripheral immunosuppression after stroke is a key pathological mechanism of SAP (Faura et al., 2021). For ICH patients, acute brain injury-induced T-cell dysfunction, lymphopenia, and weakened lung mucosal immune defense make it easier for the lungs to transform from a stable state to clinical infection progress from a stable state to clinical infection. Therefore, SAP is not only a mechanical complication, but also a clinical manifestation of immune reprogramming in the lungs after stroke.

### 5.2 Post-ICH peripheral T-cell alterations increases the risk of stroke-associated pneumonia (SAP)

From a mechanistic point of view, peripheral T-cell abnormalities after ICH are mainly manifested by decreased cell numbers, decreased effector function, and imbalance in T-cell subset polarization. Clinical studies have shown that patients with ICH can experience elevated stress hormones early after admission, accompanied by early lymphopenia and reduced cellular immune responsiveness (Liesz et al., 2013). After acute brain injury, activation of the HPA axis leads to an increase in glucocorticoids, and activation of the sympathetic nervous system leads to an increase in the release of catecholamines; both act together on immune cells in the spleen, bone marrow, lymph nodes, and peripheral blood, causing lymphopenia, T-cell hyporesponse, decreased antigen presentation, and weakened Th1-type cellular immunity. Studies on stroke-induced immunosuppression have shown that the sympathetic nervous system and HPA axis are important upstream mechanisms of immunosuppression after stroke; among them, sympathetic nerve-mediated β-adrenergic signaling is particularly closely related to increased susceptibility to infection (Mracsko et al., 2014).

#### 5.2.1 Attenuated Th1 immunity compromises pulmonary antibacterial defense

One of the key immunological bases for the development of SAP is a decrease in Th1-type antimicrobial responses (Westendorp et al., 2022). Th1 cells secrete cytokines such as IFN-γ and IL-2, which can maintain cell-mediated immunity against infection and enhance the host’s ability to clear pulmonary bacteria. Sympathetic nerve excitation after stroke can inhibit T-cell IFN-γ production, making peripheral T cells less responsive to infectious stimuli. Experimental studies by the Prass group and the studies summarized by Mracsko team all suggest that β-receptor blockade can restore T-cell IFN-γ responses and reduce the bacterial load in the lungs after experimental stroke, indicating that β-adrenergic signaling inhibits the antibacterial effect of T cells and is an important mechanism in post-stroke lung infection (Shi et al., 2018). Therefore, SAP does not occur simply because bacteria enter the lower respiratory tract; aspiration or airway colonization provides “pathogen input,” and post-stroke antimicrobial immunosuppression determines whether the host can clear these pathogens in a timely manner.

#### 5.2.2 Relative enhancement of Treg/Th2 responses limits inflammation but may reduce pathogen clearance

During the immunosuppression stage after ICH, the phenotype of peripheral T cells often shifts from a pro-inflammatory effector response to a regulatory/anti-inflammatory response, which is specifically manifested as a decrease in Th1/Th17 function, while the Treg and Th2-related anti-inflammatory pathways are relatively enhanced. Poststroke immunosuppression studies and related reviews indicate that important features of stroke-induced immunosuppression syndrome (SIIS) include lymphopenia, alterations in T-cell composition, decreased NK cells, and impaired monocyte function (Kaplan et al., 1996). This shift may be protective in the aspect of brain injury as it helps limit excessive inflammation and secondary immune damage; however, in the context of increased risk of lung infection, Treg/Th2 dominance may also represent an immunological trade-off associated with the development of SAP.

Tregs mainly suppress effector T-cell, dendritic cell, and macrophage responses through FoxP3-related transcriptional programs and molecules such as CTLA-4, IL-10, and TGF-β (Sun et al., 2026). A modest Treg response may attenuate lung injury and limit excessive inflammation, but excessive Treg dominance may also suppress Th1/Th17 antimicrobial responses, thereby delaying pathogen clearance. This dual effect is also reflected in lung infection models: in mice with pulmonary Paracoccidioides infection, Treg depletion enhanced lung CD4+ and CD8+ T-cell infiltration and increased Th1/Th17-related cytokine levels while reducing fungal burden, suggesting that Tregs can indeed limit protective effector T-cell responses in some lung infections (Felonato et al., 2012).

Therefore, the association between the relative predominance of Treg/Th2 responses and SAP following ICH should not be interpreted as uniformly detrimental. Rather, this immune shift may exert context-dependent, biphasic effects: while enhanced regulatory and type 2 immune responses may constrain excessive pulmonary inflammation and limit tissue injury, they may simultaneously compromise antibacterial host defense. Accordingly, the risk of SAP is likely determined not by Treg expansion *per se*, but by the balance between regulatory and effector T-cell responses, particularly the Treg/Th1 and Treg/Th17 axes. When Treg-mediated suppression exceeds the level compatible with an effective pathogen-clearing response, pulmonary antibacterial immunity may become insufficient, thereby increasing susceptibility to infection.

#### 5.2.3 Suppression of the Th17/γδT17 axis may weaken mucosal antimicrobial defense

In addition to Th1-mediated IFN-γ responses, Th17 cells and γδT17 cells together constitute an important component of the Th17 immune response in the lungs and play a key role in mucosal defense within the airways and lung parenchyma (Mills, 2023). Both can produce IL-17A and IL-17F, and can also release effector molecules such as IL-22 and GM-CSF under specific inflammatory conditions, thus connecting adaptive immunity, innate-like T-cell responses and lung barrier defense. In general, Th17 cells are more biased toward antigen-dependent, adaptive, and memory mucosal protection, whereas γδT17 cells exhibit stronger tissue-resident and innate-like response characteristics and can rapidly produce IL-17A early in infection, providing the initial signal for subsequent myeloid cell recruitment and local inflammatory amplification.

During pulmonary bacterial infection, IL-17A/F mainly acts on airway epithelial cells, fibroblasts, endothelial cells and myeloid cells, inducing the expression of molecules such as CXC chemokines, G-CSF, GM-CSF and IL-6, thus promoting the generation, mobilization and migration of neutrophils to the site of infection (Ye et al., 2001). This process is important for the elimination of extracellular bacteria such as *Klebsiella pneumoniae*. Among them, CXCL1/CXCL2 mainly correspond to the neutrophil chemotactic response in mouse lungs, while CXCL8/IL-8 is more commonly used to describe the inflammatory environment in human lungs. In addition to driving neutrophil recruitment, IL-17A/F can also synergize with IL-22 to enhance epithelial barrier integrity and promote the expression of antimicrobial peptides and barrier repair-related molecules, thereby improving the local defense capacity of the lung mucosa against bacteria and fungi (Liang et al., 2006).

Notably, the roles of Th17 and γδT17 cells in pulmonary immunity are clearly context-dependent. A moderate Th17 response contributes to pathogen clearance and barrier repair (McAleer and Kolls, 2011); however, when the IL-17 axis is continuously or excessively activated, the γδT17-neutrophil pathway may also lead to excessive neutrophil infiltration and increased release of proteases and reactive oxygen species, thereby aggravating alveolar epithelial damage, increasing vascular permeability, and promoting lung tissue inflammation (Manni et al., 2014). Therefore, the pulmonary Th17/γδT17 cells response is not purely protective or damaging but depends on the type of infection, stage of inflammation, cell source, local cytokine milieu, and host immune status (Rathore and Wang, 2016). Overall, Th17 and γδT17 cells perform multiple functions in pulmonary antimicrobial immunity through the IL-17A/F-epithelial/stromal cell-chemokine axis, including neutrophil recruitment, enhancement of barrier defense, antimicrobial peptide induction, mucosal memory maintenance, and regulation of inflammatory intensity.

The γδT17/Th17 response in the acute phase of ICH may be involved in BBB disruption and edema formation in the brain; but when systemic immunosuppression is enhanced, peripheral and pulmonary Th17/γδT17 functions may decline, resulting in insufficient pulmonary mucosal defense.

#### 5.2.4 T-cell hyporesponsiveness amplifies pulmonary immunosuppression through secondary myeloid dysfunction

T-cell abnormalities not only directly impair antimicrobial immunity but also indirectly promote SAP through myeloid cells. Th1-cell-derived IFN-γ is an important signal to maintain the antibacterial activation state of macrophages; when T-cell IFN-γ production decreases, the phagocytosis, reactive oxygen species production, antigen presentation, and bacterial clearance capacity of alveolar macrophages may subsequently decrease. At the same time, the increased activity of Treg/IL-10/TGF-β pathways can further inhibit the inflammatory response of macrophages and dendritic cells, reducing antigen presentation and costimulatory signals such as MHC-II, CD80/CD86, and IL-12. Therefore, in a post-stroke infection-susceptible state, weakened T-cell effector functions and insufficient antibacterial activation of myeloid cells often amplify each other, jointly weakening the lung’s pathogen clearance capacity.

In summary, SAP should not be regarded as a simple consequence of neurological dysfunction, but rather as a multifactorial high-risk event arising from the interplay of immunosuppression, neurohumoral dysregulation, and impaired antimicrobial defenses. For this reason, the prevention and control of SAP should not be limited to antibiotics and airway management, but should be more integrated into the post-stroke immune regulation framework for understanding.

## 6 SAP aggravates secondary brain injury through the lung–brain axis

### 6.1 The lung–brain axis links pulmonary inflammation to central neuroimmune dysregulation

The lung-brain axis refers to the bidirectional regulatory network between the lung and the central nervous system through neural, endocrine, immune, metabolic and microbiota pathways (Li et al., 2023). It involves the central nervous system, autonomic nervous system, HPA axis, peripheral immune system, lung immune cells, circulating inflammatory mediators, metabolites, and lung and intestinal microbiota. Research published by Hosang et al. (2022) in Nature showed that the lung microbiota can affect the central autoimmune response by regulating the state of microglia, suggesting a functional connection between the lung microbiota and the brain immune response.

In the researches of ICH, the lung–brain axis may represent a particularly important bidirectional pathway linking the primary cerebral insult to systemic complications and subsequent neurological deterioration. During the early phase of ICH, hematoma formation and degradation, damage-associated molecular pattern release, blood–brain barrier disruption, and activation of resident and infiltrating immune cells generate an intense central inflammatory response. Through activation of the sympathetic nervous system and hypothalamic–pituitary–adrenal axis, these cerebral signals are transmitted to peripheral immune organs and the lung, inducing lymphopenia, impaired T-cell and monocyte function, altered pulmonary leukocyte responses, and disruption of respiratory immune homeostasis (Shi et al., 2018). In parallel, impaired consciousness, dysphagia, weakened airway-protective reflexes, and reduced cough efficiency increase aspiration risk, whereas stroke-induced immunosuppression limits the clearance of aspirated or resident microorganisms. These neurological, mechanical, and immunological disturbances altogether create a pulmonary environment susceptible to stroke-associated pneumonia (SAP) (Hoffmann, 2017; Westendorp et al., 2022).

More critically, pulmonary infection is not merely a secondary complication of severe ICH but may actively feedback to aggravate brain injury. In experimental post-ICH pneumonia, pulmonary challenge with *Klebsiella pneumoniae* or lipopolysaccharide resulted in more severe neurological deficits, perihematomal inflammatory-cell infiltration, histopathological brain damage, neuroinflammation, and blood–brain barrier dysfunction than ICH alone, together with intensified lung injury, systemic immune dysregulation, and microbiota–metabolic disturbances (Wang et al., 2025). These findings provide direct phenotypic evidence that pulmonary inflammation can amplify secondary cerebral injury after ICH. Clinically, SAP in patients with ICH or mixed stroke populations is consistently associated with increased short-term mortality, prolonged hospitalization, greater disability at discharge, reduced functional independence, increased care dependency, and poorer subsequent recovery, all of which are likely to contribute to a lower health-related quality of life among survivors (Ji et al., 2014; Fluck et al., 2024). Pulmonary infection may transmit inflammatory signals back to the injured brain through circulating cytokines and pathogen-associated molecular patterns, endothelial activation, leukocyte trafficking, blood–brain barrier destabilization, and microbiota- or metabolite-related pathways (Adám et al., 2010; Xie et al., 2023). All these observations support a self-reinforcing pathological cycle in which ICH-induced neuroendocrine and immune dysfunction promotes pulmonary infection, while SAP subsequently enhances systemic inflammation and secondary brain injury, thereby further impairing neurological recovery.

### 6.2 SAP aggravates secondary brain injury through systemic inflammation, BBB disruption, and lung microbiota changes

After lung infection, inflammatory mediators such as TNF-α, IL-1β, IL-6, and MMP-9 released by alveolar macrophages and neutrophils can amplify central damage through cerebral microvascular inflammation and chemokine signals (Dénes et al., 2010). TNF-α and IL-1β can promote the expression of cerebrovascular endothelial adhesion molecules and leukocyte transendothelial migration, while MMP-9 activation is related to microvascular damage and BBB disruption (O’Carroll et al., 2015). Dénes et al. (2010) found in a model of chronic systemic infection combined with ischemic brain injury that chronic infection can aggravate brain damage, accompanied by MMP-9 activation and cell infiltration in brain microvessels; neutralizing RANTES can reverse this effect, suggesting that peripheral infection is not limited to the infection focus, but can aggravate central damage through changes in chemokines and brain microvascular inflammation.

The BBB is an important anatomical interface through which lung infection can adversely affect the central nervous system. In models of respiratory infection and lung injury, systemic inflammatory factors and endothelial activation increase BBB permeability and induce persistent central inflammation. Bohmwald et al. (2021) found in a human respiratory syncytial virus infection model that pathological changes in the lungs can increase BBB permeability and lead to astrocyte infection and long-term central inflammation. Chen T. et al. (2016) showed in a mechanical ventilation model that TLR4 signaling is involved in microglia/astrocyte activation and cognitive impairment in the hippocampus and cortex. More direct mechanistic evidence has recently been provided by Wei et al. (2024), who demonstrated that circulating LPS can directly target brain endothelial cells and induce CASP4/11–GSDMD-dependent endothelial pyroptosis, resulting in tight-junction abnormalities and BBB breakdown. In CASP4-humanized mice, Gram-negative *Klebsiella pneumoniae* infection similarly disrupted BBB integrity, and this effect was attenuated by selective inhibition of GSDMD in brain endothelial cells (Wei et al., 2024). Complementarily, Ma et al. (n.d.) showed in an LPS-induced severe pneumonia model that lung–blood barrier disruption occurred together with increased BBB permeability, reduced ZO-1 expression, and subsequent translocation of endogenous lung bacteria into the brain, providing a direct experimental evidence for a pulmonary-to-cerebral microbial route during severe pneumonia. In the subacute phase of ICH, I endothelial damage, astroglial foot process reaction, and MMP-related BBB imbalance already exist in the perihematomal area, so SAP-related peripheral inflammation may further disrupt BBB homeostasis and promote cerebral edema and neuronal damage.

Pulmonary microbiota dysbiosis represents an emerging and potentially important component of the lung–brain axis. Although the lungs were historically regarded as sterile, culture-independent sequencing studies have established the presence of low-biomass but biologically active microbial communities within the lower respiratory tract (Hilty et al., 2010; Charlson et al., 2011; Dickson et al., 2017). Accumulating evidence suggests that these communities can influence the central nervous system through microbe-derived products, systemic immune mediators, and metabolic signals (Serres et al., 2009). In a rat model of experimental autoimmune encephalomyelitis, Hosang et al. (2022) demonstrated that selective manipulation of the pulmonary microbiota altered the activation state of CNS microglia and modified susceptibility to autoimmune neuroinflammation. In particular, intratracheal neomycin-induced pulmonary dysbiosis shifted the pulmonary microbial community toward LPS-enriched bacterial taxa and induced a type-I-interferon-primed state in CNS microglia, which limited the recruitment and pathogenic activity of encephalitogenic T cells within the CNS (Hosang et al., 2022). Recently, Kang H. et al. (2024) showed that pulmonary microbiota-derived LPS contributed to microglial activation and neurological injury in a murine model of polystyrene microplastic exposure; depletion or modification of the pulmonary microbiota reduced circulating LPS, suppressed central inflammatory responses, and ameliorated neurological abnormalities. Together, these findings support a functional pulmonary microbiota–microglia axis in which alterations in the composition or immunostimulatory capacity of the lung microbiota can reshape the central immune microenvironment.

This pathway may be relevant during Gram-negative pulmonary infection, in which bacterial products such as LPS can enter the systemic circulation and activate circulating monocytes and macrophages, cerebrovascular endothelial cells, and microglia through TLR4-dependent signaling (Sumi et al., 2010; Banks et al., 2015). Subsequent activation of NF-κB-related inflammatory programs may increase the production of TNF-α, IL-1β, and IL-6, promote endothelial dysfunction and matrix metalloproteinase activity, and facilitate the propagation of peripheral inflammatory signals into the CNS (Dal-Pizzol et al., 2013; Galea, 2021). According to these evidences, pulmonary dysbiosis or infection may affect the brain not only through systemic cytokinemia, but also through changes in the abundance, composition, and biological activity of microbiota-derived pathogen-associated molecular patterns. In the setting of a pre-existing central inflammatory lesion, such peripheral microbial signals could further prime microglia and neurovascular interfaces, including the cerebrovascular endothelium, choroid plexus, and meninges, thereby amplifying secondary neuroinflammation (Serres et al., 2009).

When SAP occurs, the lung microecology can shift from a steady-state environment of low biomass and high diversity to the expansion of pathogenic bacteria and the enhancement of local inflammation. For ICH patients, pulmonary microbiological disturbances may indirectly affect central immunity by changing intrapulmonary antigen presentation and T-cell polarization, enhancing systemic inflammation, and reshaping the function of alveolar macrophages and dendritic cells. However, there are still few direct mechanism studies on the ICH-SAP scenario, and this part is still mainly based on inferences from the lung-brain axis and respiratory infection immunology.

### 6.3 SAP may aggravate post-ICH neuroinflammation through T-cell remodeling

From a T-cell perspective, the reverse impact of stroke-associated pneumonia (SAP) on the injured brain should not be regarded merely as the passive spillover of pulmonary inflammation. Instead, pulmonary infection may reshape the abundance, polarization, trafficking capacity, and effector state of circulating T-cell populations, thereby generating an adaptive immune component of the lung–brain axis. After ICH, activation of the sympathetic nervous system and hypothalamic–pituitary–adrenal axis can induce lymphocyte apoptosis and redistribution, suppress T-cell proliferation and IFN-γ production, and weaken Th1-type cellular immunity (Prass et al., 2003; Mracsko et al., 2014). Superimposed pulmonary infection may further intensify this immunosuppressive state by promoting persistent lymphopenia, defective IL-2 and IFN-γ production, and the expression of inhibitory receptors associated with T-cell dysfunction, including PD-1, TIM-3, and LAG-3 (Boomer et al., 2011; Bailin et al., 2019). In severe or persistent infection, sustained innate inflammatory activation may therefore coexist with impaired adaptive antimicrobial immunity, resulting in defective pathogen clearance, recurrent infection, and unfavorable clinical outcomes.

It is important to note that infection-induced remodeling of the T-cell compartment is unlikely to be uniformly immunosuppressive. Pulmonary bacterial infection activates alveolar macrophages and dendritic cells and increases local IL-1β, IL-6, IL-23, and TGF-β-related signaling, which can promote the differentiation, maintenance, or rapid activation of Th17 and γδT17 cells (Kim et al., 2013). In pneumococcal and staphylococcal pneumonia models, pulmonary γδ T cells accumulate rapidly and contribute to IL-17-dependent neutrophil recruitment and bacterial clearance, although excessive or prolonged γδ T-cell responses may also promote tissue injury (Nakasone et al., 2007; Cheng et al., 2012). IL-17A and IL-17F subsequently act on pulmonary epithelial, endothelial, and stromal cells to induce CXCL1, CXCL2, CXCL5, G-CSF, and antimicrobial peptides, thereby coordinating neutrophil production, recruitment, and mucosal defense (Chen K. et al., 2016). Consequently, SAP may produce a complex peripheral T-cell phenotype characterized by generalized adaptive hyporesponsiveness together with the selective persistence or expansion of IL-17-producing effector populations.

These changes may be detrimental after ICH because the perihematomal cerebral endothelium is already activated and the BBB remains structurally vulnerable. Human Th17 cells transmigrate across brain microvascular endothelial monolayers more efficiently than conventional CD4+ T cells, while Th17-derived IL-17 and IL-22 disrupt endothelial tight-junction organization and promote CNS inflammation (Hania et al., 2007). IL-17A can additionally induce endothelial oxidative stress and actomyosin contraction, alter the localization of ZO-1, occludin, and claudin-5, increase brain endothelial chemokine production, and facilitate lymphocyte adhesion and transendothelial migration (Wojkowska et al., 2017; Rahman et al., 2018). Experimental ischemic stroke studies further show that peripheral γδ T cells can infiltrate injured cerebral tissue and aggravate BBB damage through IL-17A, supporting the capacity of circulating γδT17 cells to participate in neurovascular injury under permissive inflammatory conditions (Xiaohong et al., 2022).

In the ICH brain, infiltrating CD4+ T cells accumulate in perihematomal regions and exert pro-inflammatory and pro-apoptotic effects on the neurovascular unit, whereas macrophage-derived IL-23 activates the γδ T-cell–IL-17A axis and aggravates cerebral edema and neurological dysfunction (Zhong et al., 2016; Shi et al., 2023). Continued T-cell receptor activation is also associated with increased cerebral T-cell infiltration, inflammatory cytokine production, cytotoxic CD8+ T-cell responses, white matter injury, and brain edema after experimental ICH (Xiu et al., 2025). Pulmonary infection may therefore act as a systemic second hit by altering the peripheral balance between immunosuppressive and IL-17-producing T-cell populations. Circulating Th17 or γδT17 cells and their cytokines may subsequently interact with chemokine gradients generated by cerebral endothelial cells, the choroid plexus, meninges, astrocytes, and activated microglia, facilitating their recruitment to CNS border compartments or perihematomal tissue. Once present within or adjacent to the CNS, these cells could amplify endothelial dysfunction, CXC-chemokine production, myeloid-cell activation, neutrophil recruitment, BBB leakage, cerebral edema, and secondary parenchymal injury.

Accordingly, SAP may establish a T-cell-associated pathological circuit in which ICH-induced neuroendocrine immunosuppression predisposes the lung to infection; pulmonary infection further remodels peripheral T-cell composition and function; and selected circulating inflammatory T-cell subsets and their mediators feedback through the damaged neurovascular interface to intensify central inflammation. However, whether the T cells entering the CNS after SAP arise specifically from the infected lung, rather than from circulating, splenic, intestinal, or other lymphoid compartments, remains unresolved and requires lung-specific fate mapping, photoconversion, parabiosis, or adoptive-transfer experiments.

## 7 The proposed ICH–T cell–SAP pathological loop

Taken together, current evidence suggests that T-cell involvement in SAP after ICH can be conceptualized as a continuous positive-feedback loop (Endres et al., 2022). In the acute phase of ICH, hematoma formation and hematoma degradation products release abundant DAMPs, activate microglia and cerebrovascular endothelial cells, trigger focal pro-inflammatory responses, and recruit γδ T cells, Th1 cells, and Th17 cells into the CNS through BBB disruption, the choroid plexus, and meningeal pathways. These pro-inflammatory T-cell subsets in turn damage the BBB through mediators such as IL-17, IFN-γ, and TNF-α, promote cerebral edema, and aggravate neuronal death. As a result, they contribute to the secondary brain injury.

As the disease progresses, persistent activation of the sympathetic nervous system and the HPA axis promotes peripheral immunosuppression, characterized by lymphopenia, impaired T-cell responsiveness, attenuated Th1-mediated antibacterial immunity, and reduced pulmonary defense capacity (Kang K. et al., 2024). During the subacute phase, this peripheral T-cell remodeling is further characterized by depletion of conventional effector T cells, relative enrichment of regulatory components, and attenuation of Th1/Th17/γδT17-associated antimicrobial responses. These changes may impair pulmonary pathogen clearance and mucosal immune defense, thereby creating an immunological background that increases susceptibility to SAP. As a result, patients enter a high-risk window for SAP.

Once SAP develops, pulmonary inflammation, microbiota dysregulation, and bacteria-associated inflammatory signals retrogradely amplify central inflammation through the lung-brain axis, further destabilize the BBB, activate microglia, and disrupt T-cell immune homeostasis, consequently aggravating secondary brain injury. In turn, worsening brain injury intensifies neuroendocrine stress responses and peripheral immunosuppression, thereby predisposing patients to more severe or prolonged pulmonary infection. In this way, ICH and SAP constitute a mutually reinforcing pathological loop in which T-cell migration, polarization, and effector function serve as major immunological relays.

This integrative framework highlights T cells as a central hub linking central neuroinflammation, peripheral immunosuppression, and pulmonary infection after ICH (Figure 2). Given their central position within this interconnected network, T cells may provide a potential target for stage- and compartment-specific immunomodulatory strategies.

**FIGURE 2:**
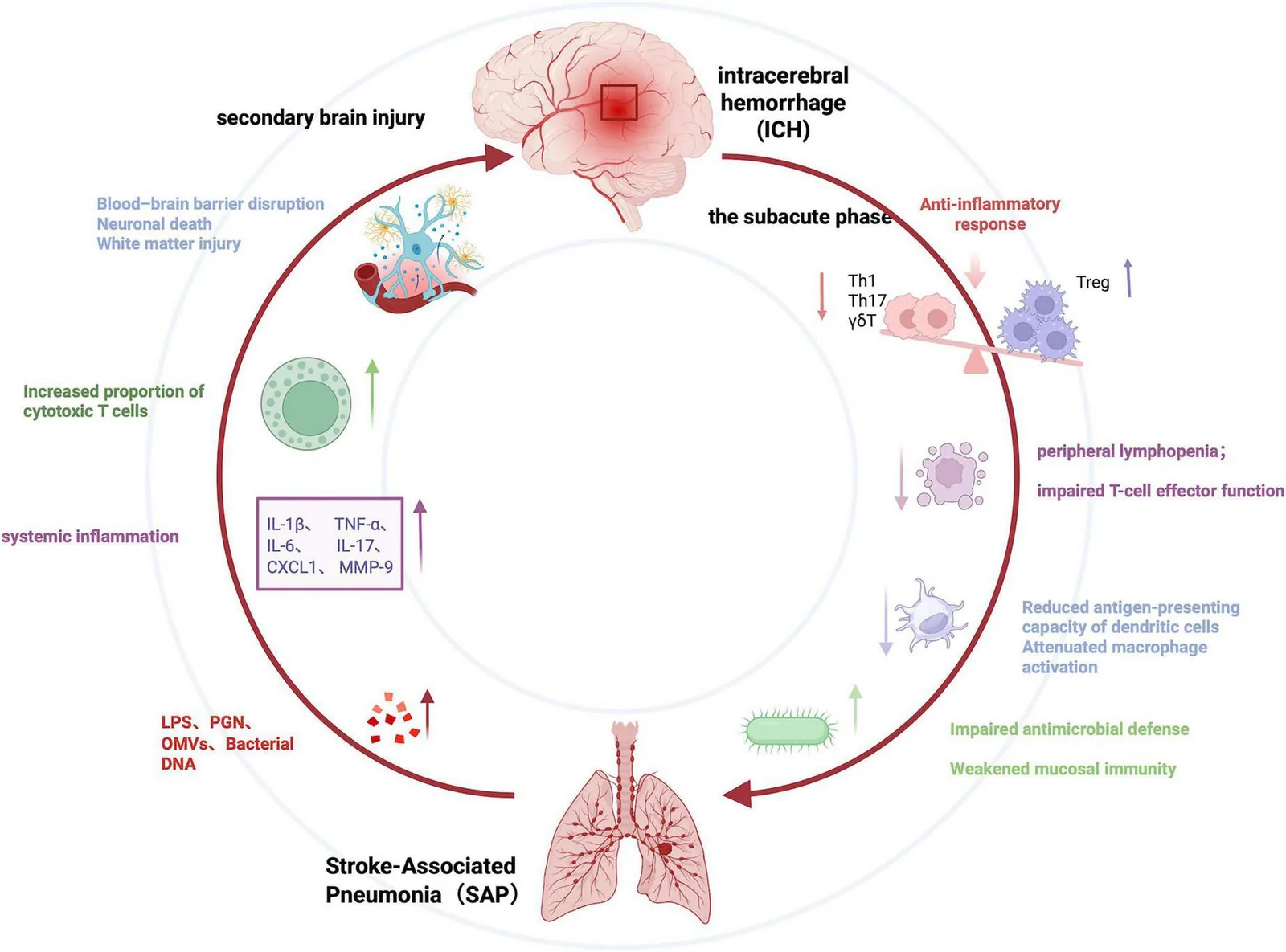
Vicious lung–brain axis cycle following intracerebral hemorrhage. During the subacute phase after intracerebral hemorrhage (ICH), the peripheral immune response shifts toward an anti-inflammatory and immunosuppressive state, characterized by reduced Th1, Th17, and γδ T-cell responses, relative expansion of regulatory T cells, peripheral lymphopenia, and impaired T-cell effector function. These alterations are accompanied by diminished antigen-presenting capacity of dendritic cells, attenuated macrophage activation, impaired antimicrobial defense, and weakened mucosal immunity, thereby increasing susceptibility to stroke-associated pneumonia (SAP). Following the development of SAP, bacterial components, including lipopolysaccharide (LPS), peptidoglycan (PGN), outer membrane vesicles (OMVs), and bacterial DNA, enter the systemic circulation and amplify systemic inflammation. The subsequent increase in inflammatory mediators, including IL-1β, TNF-α, IL-6, IL-17, CXCL1, and MMP-9, promotes cerebrovascular endothelial activation, blood–brain barrier disruption, neuronal death, white matter injury, and secondary brain injury. These processes further aggravate neurological dysfunction and reinforce a self-perpetuating lung–brain inflammatory loop after ICH. Created by the authors using BioRender.com.

## 8 Discussion

Despite substantial progress in the study of post-ICH neuroinflammation and post-stroke infection, the role of T cells in ICH-SAP remains incompletely defined. Firstly, more precise evidence is still needed regarding the spatiotemporal distribution, tissue origin, and functional conversion of distinct T-cell subsets after ICH. Future work should integrate single-cell sequencing, spatial transcriptomics, *in vivo* lineage tracing, and meningeal immune imaging to construct a comprehensive atlas of T-cell dynamics after ICH. Second, the applicability of the classic Th1-to-Th2 shift paradigm to ICH requires further clarification, particularly across different disease stages, injury severities, and complication profiles.

In addition, the lung-brain axis remains underexplored in the specific link of ICH and SAP. Most available evidence derives from ischemic stroke, traumatic brain injury, respiratory infection, or general pulmonary inflammation models. The primary purpose of proposing an ICH–SAP pathological cycle in this review is to integrate the existing direct and indirect evidence into a mechanistic framework that can be further tested, rather than to imply that every component of this proposed pathway is supported by an equivalent level of direct evidence. Future studies should establish ICH-specific experimental models of pulmonary injury to define the causal relationships among pulmonary infection, lung microbiota dysbiosis, and cerebral immune remodeling. Such studies should also determine which lung-derived signals drive the amplification of central inflammatory responses and identify the T-cell subsets that play the most critical roles in this process. Systematic investigation of candidate regulatory nodes, including lipopolysaccharide (LPS), IL-17, IL-6, type III interferons, sympathetic β-adrenergic signaling, and cholinergic anti-inflammatory pathways, may facilitate the identification of therapeutically actionable targets.

From a translational perspective, prophylactic antibiotic therapy alone does not appear to effectively improve the long-term outcomes of patients with SAP, suggesting that SAP should not be regarded solely as a microbiological complication, but rather as an immunosuppressive syndrome closely associated with the host immune state (Westendorp et al., 2015). In light of this, future therapeutic strategies should extend beyond pathogen eradication to include the restoration of immune homeostasis. Potential interventions should take into account the spatiotemporal redistribution and functional reprogramming of distinct T-cell subsets after ICH. During the early phase, excessive inflammatory responses mediated by γδ T cells and Th1/Th17 cells may need to be restrained, whereas during the recovery phase, Treg-mediated resolution of inflammation and blood–brain barrier (BBB) repair may be therapeutically beneficial. At the same time, efforts to limit inflammation-driven secondary injury to the central nervous system should be carefully balanced against the potential risk of infection associated with excessive immunosuppression. Only by achieving a more precise balance among the control of central inflammation, preservation of peripheral immune competence, and management of infection can the vicious cycle linking ICH, T-cell dysregulation, SAP, and aggravated neurological injury be effectively disrupted.
